# Development of myocarditis and pericarditis after COVID‐19 vaccination: Comment

**DOI:** 10.1002/clc.23986

**Published:** 2023-02-20

**Authors:** Amnuay Kleebayoon, Viroj Wiwanitkit

**Affiliations:** ^1^ Private Academic Consultant Samraong Cambodia; ^2^ Department of biological science Joseph Ayobabalola University Ikeji‐Arakeji Nigeria

Dear Editor,

We would like to share ideas on the publication “Development of myocarditis and pericarditis after COVID‐19 vaccination in children and adolescents: A systematic review.^1^” To conduct a thorough literature search, Fatima et al. used three databases: Cochrane, MEDLINE/PubMed, and EMBASE from the beginning through March 2022.^1^ The review comprised a total of three case reports, four case series, and six observational studies.^1^ Myocarditis and pericarditis in children and adolescents following the COVID‐19 vaccinations were more frequently seen in males and after the second dosage of Pfizer, according to Fatima et al.^1^ Although the incidence was generally modest, Fatima et al. came to the conclusion that doctors should consider myocarditis and pericarditis as plausible diagnoses when dealing with young patients who have a history of receiving vaccinations and who present with suggestive signs.^1^

It is often challenging to identify the precise clinical relationship because there is minimal clinical information available on the health and immunological status of vaccine recipients before injection. Although comorbidities do exist, they are hardly discussed in published studies. Due to a paucity of information on the health and immunological status of vaccine recipients before to injection, establishing the precise therapeutic relationship may occasionally be challenging. Comorbidities in the patient could have made things worse. A major worry that could affect clinical outcomes is the potential for concomitant dengue infection.^2^ Finding the precise clinical connection can be simple because there is little information available about the health and immunological state of vaccine recipients before inoculation.

The patient's comorbidities may have played a role in the problem. It can be difficult to pinpoint the precise clinical relationship at times. Additionally, research has shown that the genetic diversity of the inheritors affects immune responses. According to one study,^3^ a person's genetic makeup may influence how their immune system responds to immunizations. Before making a decision, a test must be conducted. More research is necessary to rule out any additional aggravating factors, such as comorbidity or an underlying pathological condition. It is important to consider any current or previous asymptomatic COVID‐19 infections. It might be a good idea to take the scenario into account given how special it is.^4^

## CONFLICT OF INTEREST STATEMENT

The authors declare no conflict of interest.

## Data Availability

Data sharing not applicable to this article as no datasets were generated or analyzed during the current study.
